# Lessons learned through the 20-year development of a national fatal drowning database in Australia

**DOI:** 10.1186/s12889-023-16392-2

**Published:** 2023-08-07

**Authors:** Amy E Peden, Stacey Willcox-Pidgeon, Justin-Paul Scarr, Richard C Franklin

**Affiliations:** 1 Royal Life Saving Society – Australia, Broadway, NSW 2007 Australia; 2 School of Population Health, Faculty of Medicine and Health, UNSW Sydney, Kensington, NSW 2052 Australia; 3 College of Public Health, Medical and Veterinary Sciences, James Cook University, Townsville, QLD 4811 Australia

**Keywords:** Epidemiology, Risk factor analysis, Death registry, Drowning, Water safety, Health policy, Leadership, Children, Alcohol

## Abstract

**Background:**

Co-ordinated, evidence-based policy and programmatic efforts are needed to respond to complex drowning prevention problems. Comprehensive, current, and robust data are vital for agenda setting, burden and risk factor identification, intervention design and evaluation, as well as setting policy. We aim to record methods used in, and identify impacts of, the development of a national fatal drowning database (NFDD) in Australia, including lessons learned across research, policy, and practice.

**Methods:**

We employ a case study method using process mapping and document review to explore the evolution, drivers and impacts of the NFDD. We analyse methodological approaches including those relating to data definitions, drowning case collection, and management, as well as tracking the various outputs of the NFDD. We describe a development timeline that presents impact of drowning prevention policy, and research agendas on database development, and research investments more specifically.

**Results:**

Our study identified that the collected variables grew 20-fold from 2002 to 2022, reaching 259 variables, and 5,692 unique cases of fatal drowning. The NFDD employs data triangulation methodology, combining keyword and targeted searches of coronial files, media report monitoring, and organisational data provision. Database development is influenced by the Australia Water Safety Strategy, policymaker and practitioner-initiated research agendas, and identification of knowledge gaps. We identified numerous outputs spanning publications, media, intervention development, and legislative submissions.

**Conclusion:**

A comprehensive and robust NFDD informed by policymaker and practitioner input can enhance surveillance, policy, and intervention development for drowning prevention. Employing mixed data collection and validation methods can supplement weaknesses in official data sources. There is a need for the NFDD to continue to evolve in its application while maintaining rigorous case identification and data quality assurance processes. Despite significant investment, the outputs and influence on drowning prevention practice in Australia has been extremely valuable and contributed to sizeable reductions in Australia’s fatal drowning rate.

**Supplementary Information:**

The online version contains supplementary material available at 10.1186/s12889-023-16392-2.

## Background

Drowning is a significant cause of injury-related mortality and morbidity [1]. Globally, it is estimated that 295,000 people (95% confidence intervals: 284,493 to 306,187) died from unintentional fatal drowning in 2017 [2], though such estimates exclude transportation and disaster related drowning due to a narrow definition of unintentional drowning based on the International Classification of Diseases (ICD) coding framework [3, 4]. Nationally, the underreporting of drowning in Australian Bureau of Statistics (ABS) data has been estimated at 40% [4]. Similarly, using ICD coding frameworks around location and activity prior to drowning provide limited information from which to identify drowning risk factors and develop prevention interventions.

The World Health Organization (WHO) recommends advancing drowning prevention through data collection and well-designed studies, as one of four strategies, alongside six selected interventions to prevent drowning at a community or national level [5]. Comprehensive, country-level data on drowning can address current shortcomings around accuracy and completeness of data estimates, and limited risk factor identification.

Although there is a long history of drowning prevention and water safety in Australia, Australia is not the only country working on a National Drowning Database which includes detailed information about the drowning incident, with some cross country analysis using national databases having been published in the last few years, such as the Australia, Canada, and New Zealand collaboration [6–8]. One of the lessons the authors discuss in these papers is the need for better definitions that are internationally consistent [6]. Another drowning database development is in the United Kingdom [9], while this study focused on a subset of total drownings (2012–2019) it undertook an audit and provided recommendations associated with data capture procedures.

To address this gap in the literature, we focus on the National Fatal Drowning Database (NFDD) of the Royal Life Saving Society – Australia (RLSSA) which has been collating, analysing, and communicating cases of unintentional fatal drowning for 20 years. Data are collated and analysed in the annual Royal Life Saving National Drowning Report, to increase awareness of drowning risk and communicate risk reduction strategies [10]. Data also inform the development of the Australian Water Safety Strategy (past and present), including the identification of goal areas and measurement of progress in reducing drowning against key indicators [11].

Within a period of rapid transition in the technology and media landscape, as well as the growth of the field of drowning prevention [12], the database has evolved over 20 years of data collection, analysis and reporting. In the current study, using a case study approach [13], we describe methodological approaches used to develop, populate, and maintain the NFDD and aim to identify and document impacts and insights of this approach for researchers, policy makers, and practitioners as well as helping other countries develop similar registries.

## Methods

This paper takes a case study approach [14] to describe the development, implementation, and use of the NFDD. Within our case study approach several methods have been used: process mapping; process tracing of impacts; and document review. Firstly, we used process mapping to identify and document the formal and informal structures and processes applied in the delivery of the NFDD [15]. The processes of case identification, verification, collation, and analysis of drowning data via the NFDD were mapped. We then use process tracing, i.e., a data analysis method for identifying, validating, and testing causal mechanisms within case studies in a specific, theoretically informed way [16], to trace the impacts and outputs of the NFDD. To complement the process tracing, we reviewed a range of documents including peer-reviewed literature and industry reports to analyse the outputs and impacts of the NFDD.

## Results

### Background

The NFDD was first developed in 2002 to address the paucity of detailed drowning data in Australia. Originally the Australian Bureau of Statistics (ABS) had a drowning flag (i.e., an identification field for all drowning-related cases) that was added to the annual cause of death data they produced. However, this ceased in 2002 [17]. In 2000 the National Coronial Information System (NCIS) was developed to collect all sudden and unexpected deaths in Australia which were investigated by the coroner. This online repository of coronial records allowed for the electronic access of coronial records, something that was previously difficult and time consuming with the need to personally visit all coronial offices. From 2000, Royal Life Saving invested in the use of a media monitoring service to both track advocacy and campaign impact, as well as identify cases of drowning in the media. The NFDD was used to consolidate all drowning cases identified via the NCIS, media and other sources (such as Child Death Review and lifesaving agencies) as detailed below.

### Process mapping

We mapped the processes employed and identified a range of activities such as case identification, verification, collation, and analysis. In many cases overlapping and developed iteratively over the database’s 20-year lifespan.

### Data sources

**Fig. 1:**
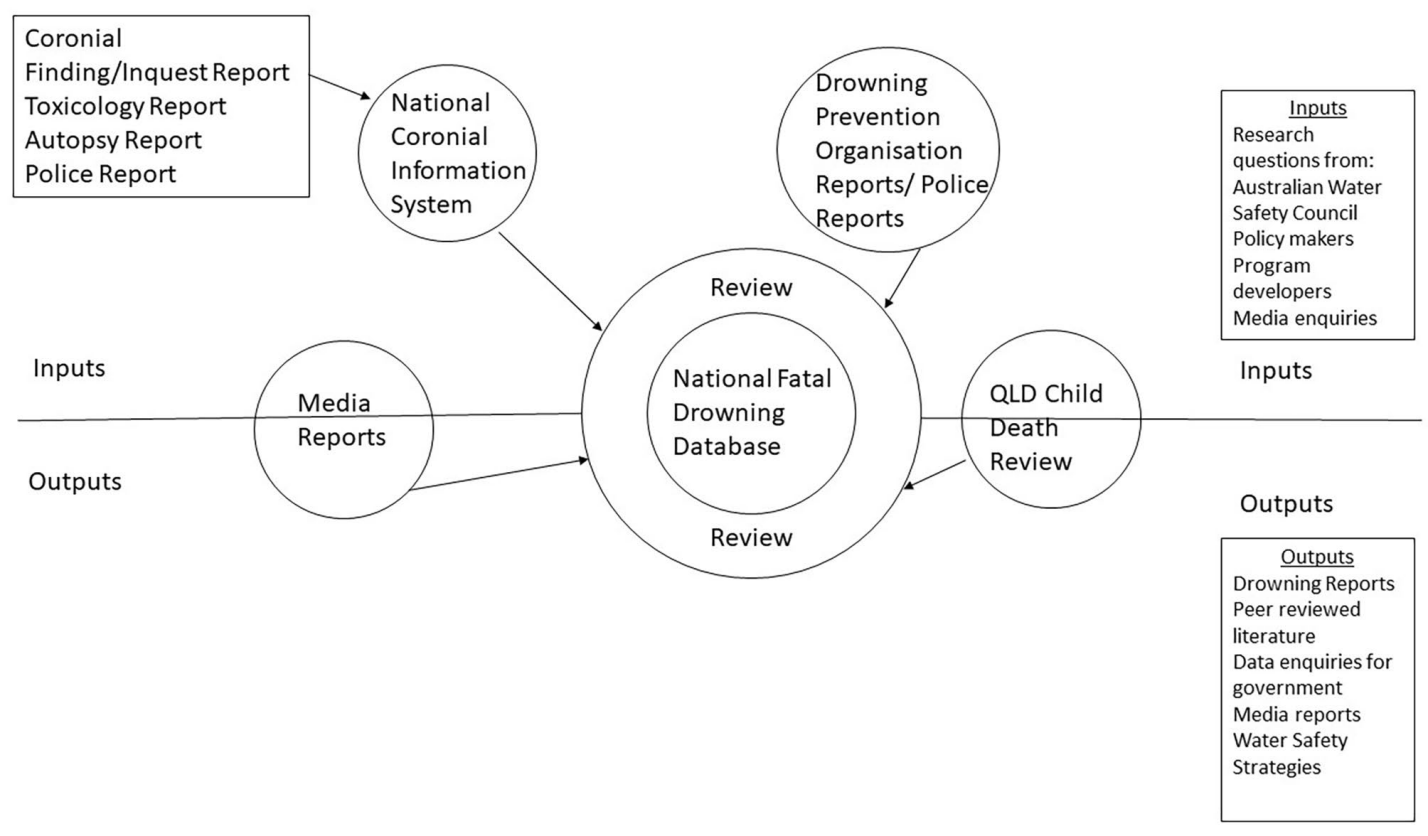
Pictorial depiction of data sources which feed into the National Fatal Drowning Database, as well as related outputs. Note media reports and QLD Child Death Review are both inputs to the NFDD but also act as outputs of the NFDD.

There are a range of data sources which feed into the NFDD (Fig. 1). Each has an important role in case identification, verification, and data collation. It must be acknowledged that not all data sources are available for each incident and each approach has its own strengths and limitations.

#### Media reports

Year-round media monitoring is conducted using an external media monitoring company. This company searches for pre-defined keywords across print, broadcast (radio and television) and online media reports and social media posts. Daily digest emails of relevant media coverage are sent to RLSSA staff. Preliminary data on a relevant case that is believed to fit the inclusion criteria are entered by RLSSA researchers into an SPSS media monitoring database. Preliminary free text data fields comprise age (or age range if specific age unknown), sex, date of incident, date of death, geographical location of incident, aquatic location of incident, activity prior to drowning, outcome (i.e., fatal, non-fatal, missing, N/A) notes and a link to the media article for future reference). Cases of non-fatal drowning are initially entered into the media monitoring database to monitor regarding reporting of patient outcome in the media and to cross-reference in case of a fatality against the NCIS.

Keywords used to identify cases of drowning reported in the media can be found in Table 1. It should be noted that, although near-drowning is no longer accepted terminology to describe a non-fatal drowning [18], its use persists in media reports.

**Table 1:** Keywords used to identify cases of drowning reported in the media

Drowning, Drowned, Drowns when mentioned within 10 words of: Beach, Death, Die, Died, Pulled From Water, Pulled Out Of The Water, Swimming Pool, River, Ocean, Surf, Lake, Dam, Aquatic Centre, Water Slide, Inflatable, Aquatic Facility, Swim School, Creek, Flood, Flood Water, Flood Waters, Swim Centre, Swimming	
Body **when mentioned within 20 words of**: Pulled From Water, Pulled From The Water, Pulled Out Of The Water	
Water Police, Marine Area Command **when mentioned with**: Search, Rescue, Water Search, Water Rescue	
Caught in a rip, Swimming Accident, dies while swimming, dies in floodwater, Died in Floodwater, swift water rescue	
Flood, Floodwater, Floodwaters, Flash Flooding, Flood Water **when mentioned within 30 words of**: Died, Drown, Drowning, Dies, Dead	
Boating Accidents, Boating Accident **when mentioned within 20 words of**: Death, Drowning	
Ferry Capsizing, Boat Capsizing, Rock Fishing Rescues, Rock Fishing Rescue	
Inquests Findings, Coronial Findings **when mentioned within 30 words of**: Rivers, Swimming Pools, Beach, Ocean, Surf, Lake, Dam, Swimming Pool, Aquatic Centre, Water Slide, Inflatable, Aquatic Facility, Swim School, River, Creek, Flood, Flood Water, Flood Waters, Swim School, Swim Centre, Swimming	
Bravery Awards, Bravery Award **when mentioned with**: Royal Life Saving, Water Rescue, Water, Ocean	
Near Drowning, Near-drowning **when mentioned within 20 words of**: Serious Injury, Brain Injury, Hypoxia, Paralysis, Critical Condition	

#### Drowning prevention organisations

Data are sourced from drowning prevention organisations across Australia at the national and sub-national level, such as Royal Life Saving, Surf Life Saving and maritime safety organisations. Surf Life Saving provide additional data on coastal incidents (both fatal and non-fatal, drowning, and other cause incidents) through their Incident Rescue Database (IRD) and their own media monitoring and NCIS searches [19]. Maritime safety organisations provide additional insights regarding identification and verification of boating and watercraft incidents, including relevant risk factors.

#### Child death review

Child Death Review Teams at the sub-national level are a governmental function which reviews the deaths of children (that is young people aged 0–17 years) [20]. This includes maintaining a registry of child deaths, identifying the cause of death, conducting research, and making recommendations for legislation, policies, practices, and services to prevent or reduce the likelihood of child deaths. RLSSA cross-references collated cases of child drowning with child death review teams from Queensland to confirm cause, intent, and relevant risk factors.

#### National coronial information system (NCIS)

The NCIS is an online repository of all sudden and unexpected deaths investigated by a coroner in the eight states and territories of Australia and in New Zealand. Drowning is considered a sudden and unexpected death and therefore must be investigator by a coroner. The NCIS, accessible to researchers via ethical approval, comprises information from the investigation conducted by a coroner into the death of an individual [21]. This information comprises data fields for an individual’s NCIS case, as well as attachments comprising any combination of the following: coronial finding (or inquest report), autopsy report, toxicology report, police narrative of circumstances.

A police report will detail the narrative circumstances leading up to the incident. Toxicology provides blood and urine testing results for illicit and prescription medications including type and concentration, as well as alcohol content. Autopsy report will detail characteristics of the individual such as sex, heigh and weight, as well as the presence of acute injury or medical condition which occurred during the drowning incident, as well as any evidence of pre-existing disease or injury. An internal autopsy can be opposed, resulting in findings from an external examination only.

RLSSA researchers gained access to the NCIS in 2001. This allowed the identification and collation of drowning cases from all jurisdictions via the one portal. Prior to this, cases were captured from individual state and territory coroners’ courts. NCIS access allows drowning cases from all states and territories in Australia to be identified through a mix of searches on fields or attachments (should the case be closed and documentation attached) (see Table 2). In more recent cases which remain open within the coronial system and thus show limited information, media reports of an incident are matched to the case and used to supplement missing information in the NFDD record for the case.

**Table 2:** Example National Coronial Information System (NCIS) Search terms

Search type	General classification	Detailed classification
Cause of death search	Cause of death	Drown; Hypox; Immers; Ship; Submer; Water
Location search	Countryside	Area of still water; Base of precipice, cliff; Beach, bank, shore of body of water; Drain, channel; Hot spring, thermal pool; Large area of water; Stream of water
Location search	Home or dwelling	Yacht, boat, bathtub, spa, swimming pool
Location search	Other place of occurrence	Watercraft; Wharf, pier, jetty
Location search	Sports and athletics area	Public swimming centre
Mechanism of injury search	Threat to breathing	Drowning, near drowning
Activity search	Vital personal activity	Washing, showering, bathing

### Inclusion and exclusion criteria

Drowning is defined as the process of experiencing respiratory impairment due to submersion or immersion in a liquid, with outcomes comprising death (fatal drowning) or non-fatal drowning (with or without morbidity) [18]. There is currently debate about the need for a separate classification system for non-fatal drowning [22]. The NFDD focuses on fatal drowning which is unintentional or of undetermined intent including those where the primary cause of death may be something other than drowning, but where drowning was a contributory cause of death [4]. Drowning which is deemed to be intentional (suicide, homicide, assault, infanticide) are currently excluded. A separate data process is employed for the collection and collation of non-fatal drowning data.

The NFDD includes cases where drowning is determined to be either the primary or a contributory case of death by a coroner, as reported by the NCIS. Cases are included based on a combination of cause of death text and ICD coding (i.e., case identified if cause of death text included drowning, submersion, or immersion, and/or ICD codes were any drowning code not limited to W65-74). By not limiting case inclusion to those coded with ICD codes W65-74, this allows for the identification and inclusion of drowning deaths due to water transport and disasters incidents. Cases of suspected drowning, where the body is unrecovered, are included if the coroner rules the cause of death is likely to be drowning given the circumstances of the incident, however this is rare. Each person who drowned in a multiple fatality event (i.e., a single incident with multiple drowning deaths) is included as a separate case.

The NFDD includes cases of drowning of Australian residents or international visitors to Australia, provided the drowning incident occurred in Australia, including in Australia’s territorial sea which extends up to 12 nautical miles offshore [23]. Drowning incidents involving a foreign national which occurred in Australia but resulted in the drowned person being repatriated to their home country for treatment, prior to death are included if the death is recorded on the NCIS. Conversely, drowning incidents of Australian residents which occur overseas but result in Australian residents being repatriated for treatment in an Australian medical institution who then subsequently die are excluded.

### Database design and management

Drowning case data identified from the above sources are transferred to the NFDD which is housed within an SPSS database [24]. Each year, cases are collated directly into a new SPSS file that retains the overarching NFDD variable and coding structure. After finalisation of case numbers for that year, the dataset is imported into the overarching NFDD and new data categories are added to the calendar and financial year data categories. Cases are labelled by their status in the NCIS (i.e., open or closed), with open cases checked regularly against the NCIS. When a case is closed within the NCIS, the case record in the NFDD is updated to finalise data variables and the case status is changed to ‘closed’. This typically means the record is no longer edited, unless additional project specific variables need to be retrieved for the case or unknown variables are updated in the NCIS.

### Data entry, cleaning, coding, and definitions used

Data are entered manually, using pre-defined sub-categories for variables wherever possible to reduce typographical inconsistencies which impact analyses. Other free text fields allow for the inclusion of a short narrative sentence detailing key points. The core variables, i.e., those checked and collected (if available) for each case, are shown in Table 3. Additional project-specific variables are collected as needed. A complete list of database variables can be found in Supplementary Table 1. Variables are coded using a separate definitions and coding manual which is regularly updated to reflect changes in the database [25]. This manual ensures consistency and forms the basis for inducting new staff into the update and management of case inclusion and cleaning in the NFDD.

**Table 3:** National Fatal Drowning Database predefined sub-categories and core variables

Sub-Category	Variable name
Case information	NCIS Number
	State of Death
	State or Territory of Coronial File
	Fin Year Numeric
	Status of Case
	Checked Date
Demographics	Sex
	Age in Years
	Age Groups by 5-year bands
	Born overseas YN
	Country of Birth
	Australian resident or overseas visitor
	Time in country
	Visa
	Aboriginal and Torres Strait Islander (ATSI) Status
Cause of death	Medical Cause of Death
	Other Medical Causes Identified
	Underlying Medical Condition (Yes/No)
	Medical Condition Known
	Type of Underlying Medical Condition
	Medical condition contributed
Date and time	Date of Death
	Date of Incident
	Day of week
	Season of Incident
	Time of Incident
	Time Coded Bands (early morning, morning, afternoon, evening)
Location	Location Detailed
	Location Name
	Location Specific
	Location (Incident) Postcode
	Incident Road Address
	Incident Latitude
	Incident Longitude
	Visitor Status
	Resident Address
	Remoteness Classification of Time Location (Incident) Post Code
Activity	Activity Detailed
	Activity Specific
	RLSSA Activity Coded
	Boating or watercraft
	Type of boat or watercraft
	Multiple Fatality Event
	Number of victims per multiple fatality
Risk Factors	Lifejacket
	Swimming Ability of Deceased
	Alcohol (Yes/No)
	Blood Alcohol Content (BAC)
	Drugs (Yes/No)
	Type of Drug Known
	Drug Legal (Yes/No)
	Drug Type
	Drug Type and Reading

### Data governance, security and ethics

Strict data governance and security measures are in place given the sensitive nature of this data. Every five years, RLSSA revises and confirms an ethics approval process with the Victorian Department of Justice and Community Safety Human Research Ethics Committee (JHREC) (CF/07/13,729; CF/10/25,057; CF/13/19,798). As part of the process of gaining national ethics approval via the JHREC, a separate ethics approval process is undertaken with the state of Western Australia. In accordance with ethical agreements, data must be reported in aggregate format to minimise identification. In a practical sense, this typically precludes data counts of < 5 from being reported. De-identified data are stored on a password protected remote server housed internally at RLSSA which is backed up daily. Only limited numbers of pre-approved researchers from RLSSA have access to the NCIS and the NFDD. Media and researchers can make enquiries of the database via email which allows for the NFDD to be used for information and advocacy purposes while maintaining the accuracy and ethical constraints around data usage via aggregate reporting.

### Resourcing and funding

The NFFD was developed and maintained by staff at RLSSA. Tasks associated with the NFFD are included in work plans and tasks descriptions of research staff. IT staff assist with data security and stability of the software used. Funding for the NFDD is received from the Australian Government by RLSSA as one of a range of projects and ongoing functions of the organisation.

### Database structure

The NFDD has grown over time, both in number of cases included but also in number of variables recorded. At the time of writing, the NFDD is in its 21st year of data collation for the 2022/23 financial year (July to June). As such we report 20 years of data in the following section. At the present time, the NFDD spans 20 complete financial years and 5,692 cases, 83% of which are closed in the NCIS. Cases which have occurred more recently are more likely to be open (i.e., latest financial year) and thus details subject to change pending the outcome of the coronial process increases.

**Fig. 2:**
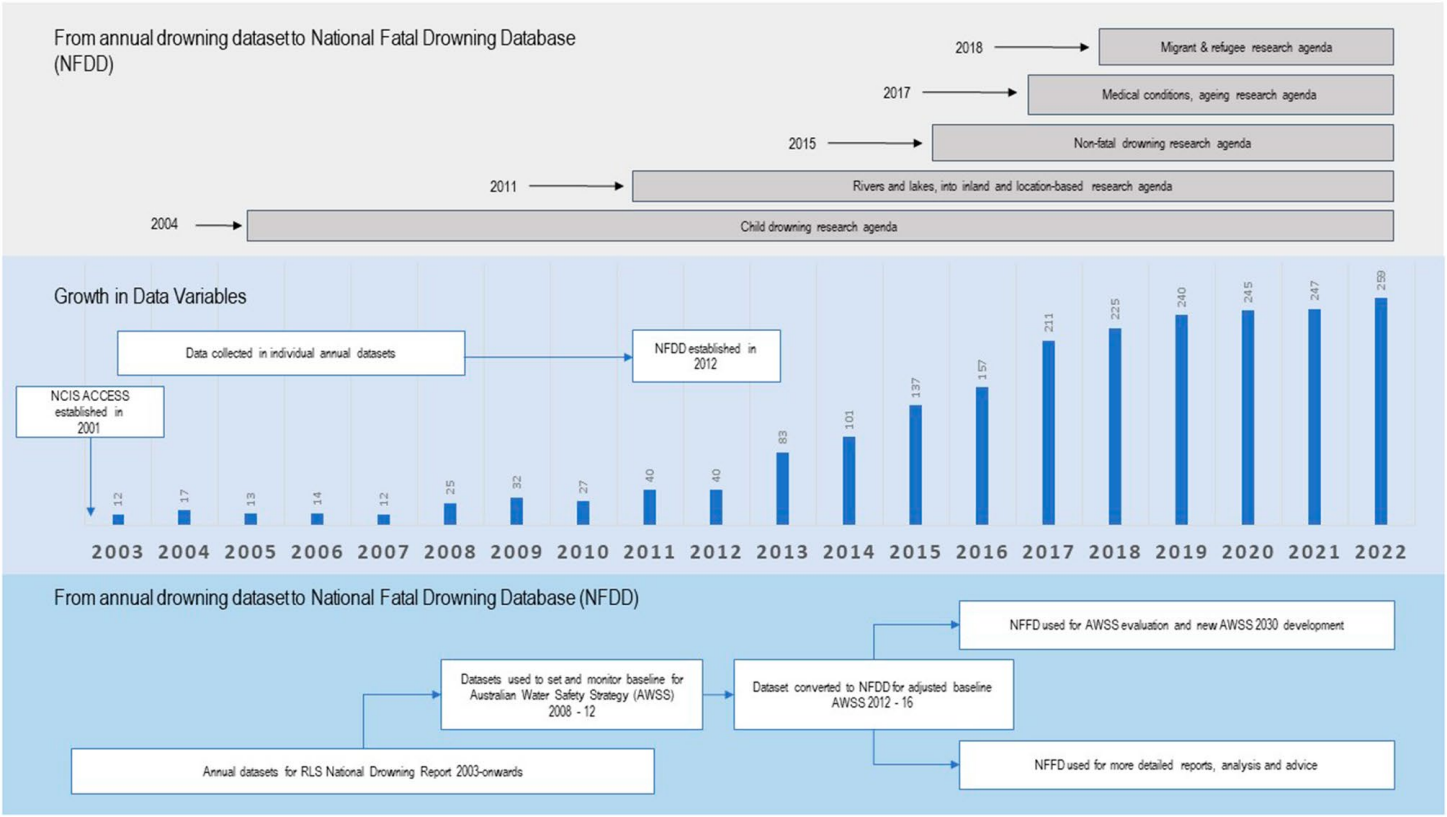
Expansion of the National Fatal Drowning Database 2003–2022

With respect to data variables these have grown in tandem with an expanded research focus. Variables have morphed from a core minimum set of variables (n = 12) used in early annual drowning reports to a possible 259 variables in 2022. Pivot points in the NFDD’s development have occurred based on specific research needs or questions which have necessitated the addition of new variables. These pivot points based on different research agendas, and the resultant expansion in variables recorded within the NFDD are depicted in Fig. 2.

### Outputs and impacts of the NFDD

There are a range of outputs the NFDD has contributed to. These include annual national drowning reports and associated media coverage, National Water Safety Strategies, industry reports, peer reviewed publications, advocacy campaigns, and the provision of information in response to requests and formal submissions. We have collated the categories of outputs and associated impacts in Table 4.

**Table 4:** Example outputs and impacts of the NFDD grouped by type

Timeframe	Type of output	Purpose	Summary of impact(s)
2002*-ongoing (Annual)	National Drowning Report (n = per annum)	To report previous financial year’s fatal (and non-fatal) drowning trends.	Used as a mechanism to engage government, other key stakeholders and media
2002-ongoing	National Water Safety Strategy (n = 3) Rural and Remote Water Safety Plan (n = 1)	A national strategy to reduce drowning in Australia that identifies key areas to focus drowning prevention efforts based on the drowning statistics	Goal alignment across sector, tracking of progress, political engagement
2002-ongoing	Data shared and presented within sub-national water safety plans (state and territory level) and local water safety plans	State/territory and/or local community level documentation of drowning burden, risk factors and opportunities for prevention	Local ownership of, and action on, issue, alignment across sectors
2002-ongoing	Industry Reports	To provide an in-depth analysis of drowning deaths to inform drowning prevention policy and practice	Media focus; political engagement; additional funding; development of advocacy campaigns
2017-ongoing	Symposium Declarations (n = 3)	To host issue focused symposium which produce an action statement for sector to align with	Focuses sector on a topic of need, action statement provides cross-sector buy in and alignment on a priority topic.
2002-ongoing	Peer-reviewed publications (n > 40)	To investigate key research questions and address gaps in the evidence base regarding drowning epidemiology, risk factors and prevention strategies	Peer-reviewed publications published that use the NFDD as the primary source of data. Has improved the evidence base, raised awareness of drowning and the NFDD across a range of journals and disciplines domestically and internationally.
2002-ongoing	Advocacy campaigns/programs	To identify priority populations or issues where campaigns or education programs are needed. To provide data insights to enhance campaign strategies.	Range of national programs and campaigns developed including annual Keep Watch child downing prevention campaign, Respect the River program, Make the Right Call, Grey Medallion, Summer Drowning Toll
2002-ongoing (informed by NCIS data)	Information requests (n > 230 to date)	To respond to requests for information on drowning from media, researchers, government and the general public	The NFDD has allowed information requests to be answered more efficiently which has resulted in media coverage of the issue which in turn has generated more requests for data
2005-ongoing	Submissions (such as information on fatal drowning risk factors to support the coronial process and information to government regarding proposed legislative changes)	To provide evidence-based information in response to requests for information regarding potential legislation or strengthening of existing legislation	The NFDD has allowed for data to support arguments around need for legislation or legislative effectiveness in instances of legislative review such as pool fencing legislation and legislative review, and mandatory lifejacket wear legislation for boaters and rock fishers – all at the sub-national level

*Drowning Reports pre-date this period, indicates where NFDD used in its creation

## Discussion

Drowning is a significant cause of injury-related harm in Australia [11]. Investment in, and ongoing commitment to, the development, maintenance, and expansion of the NFDD has resulted in a powerful and widely impactful resource to guide drowning prevention efforts across Australia. Although not entirely attributable to the NFDD and associated outputs, it is important to note Australia’s national fatal unintentional drowning rate has declined by 28% since baseline (2004/05-2006/07) [11], which we believe has been assisted by a greater understanding of who is drowning, where and why. Given the sizeable economic cost of unintentional drowning deaths to the Australian economy [26], investment in a means to identify risk factors and track the impact of drowning prevention interventions, is imperative.

In this study, we have outlined the rigorous data triangulation method used in case identification and documentation, as well as the expansion of the database in response to emerging research priorities. Finally, we have tracked the many and varied direct outputs, and impact of those outputs, since the NFDD’s inception. We reflect here on some of the lessons learned across the first 20 years of the NFDD, a period which has seen rapid change in information provision, the media landscape, and technology.

Coronial data, via ethical access to the NCIS, is the data gold standard and in large part, underpins the NFDD. The NCIS is a rich source of data geared towards prevention, although the speed with which data become available is subject to the pace of processes within the coronial system. While core demographic details such as a person’s age and sex (as well as the state or territory of the death) are recorded almost immediately when a death is entered on the NCIS, other information, such as location and activity, are often not updated until a coronial investigation is completed and the case is closed. As such, using media reports as part of a data triangulation approach, can fill these gaps and provide an immediacy of data useful for advocacy purposes, such as the RLSSA Summer Drowning Toll [27] website which is updated daily over summer to provide journalists with up-to-date data to enhance public awareness during a high-risk period. The goal is for all cases identified via data triangulation methods to be matched and cross-referenced to cases in the NCIS.

From the other direction, this triangulation approach addresses some of the known weaknesses with the use of media reports, such as a bias towards child drowning and emotive cases such as rescuer drowning and multiple fatality events, to the detriment of the identification of drowning among the elderly and in more geographically isolated locations [28]. Being aware of such limitations and verifying cases from media with a gold standard data source such as the NCIS improves rigour [6, 9].

One of the key impacts of the NFDD has been the anecdotal increase in reporting of drowning incidents by the Australian media, due in part to the NFDD providing the ability to quickly and easily respond to media requests for information, coupled with timely media-facing advocacy activities such as the Summer Drowning Toll. Systematic collation of media coverage of drowning incidents afforded by media monitoring, as used in data triangulation for the NFDD, can also provide an opportunity to audit the manner in which drowning incidents are being reported, including the provision of water safety and drowning prevention information to readers, which has yet to be done on a national basis [29].

More broadly, the impacts of the NFDD are wide ranging and, at times, hard to quantify. One of the key lessons learned over the lifespan of the NFDD has been the need to track outputs and impacts from the start. Not only is this useful from an organisational perspective, but it can be helpful as a means of justifying resourcing and securing additional investment. Conversely, the increased investment in the NFDD corresponded with an uplift in investment in research within the organisation. As the NFDD grew, so too did its uses, with additional research questions able to be asked of the database to support emerging research agendas.

Another lesson across the lifespan of the NFDD has been the technology required to support an ever-expanding database, both sadly in the number of cases added each year, and the number of variables potentially recorded for each case. As variables grew, a focus on the core minimum dataset required to annual analysis in the National Drowning Report has been key. Ensuring consistency in application of coding of variables has been an important part of the NFDD development. Having tools for training staff in the integrity of the database as staff change over the years has been a core part of this approach.

Intentional drowning is an issue which has been growing in prominence among the drowning prevention community [30, 31]. Although it could be argued to be outside of the scope of mainstream drowning prevention efforts, in practice lifeguards and lifesavers are responding in cases of intentional self-harm around water [30]. Risk factors and prevention strategies, such as brief interventions and restricting access, have parallels across unintentional and intentional drowning [32]. Within the required ethical constraints and processes, there is a need for future iterations of the NFDD to include intentional drowning deaths to provide further insights into the wider prevention of drowning.

As the NFDD continues to expand, investment in data storage and technology solutions will be an important consideration. Similarly, in a contested landscape, including the speed with which media operate, it will be important to develop a means for those outside of the organisation to interrogate the database in an ethically appropriate way themselves. This will improve responsiveness, with caveats regarding data accuracy, and also likely further enhance the reach and impact of the database. Sharing of data is a core benefit of the NFDD and an important role that RLSSA plays in data stewardship.

This study reflects the processes undertaken and impacts from the 20-year development and evolution of the NFDD. It must be noted that these reflect the experiences of one organisation, and potentially may differ from the experiences and processes applied by other organisations in Australia. The NFDD benefits from wider and long-term investment in the health and justice systems, specifically through coronial processes, health data capture and the NCIS itself. Therefore, the processes adopted by the NFDD may not be entirely transferable to other country contexts. It is also not an exhaustive documentation of all outputs and impacts of the NFDD which are too numerous to list and difficult to fully track.

## Conclusions

Timely and accurate data on drowning are important for a range of reasons, including research, advocacy, to influence policy change and as a mechanism for monitoring progress against stated goals in drowning reduction. Creating and maintaining high quality databases such as the NFDD, require a rigorous approach to case identification, validation and coding, as well as significant and ongoing resourcing. However, the outputs and impacts can be wide-ranging and highly impactful. In future, revitalisation of technology may be required to house an expanding dataset, as well as innovative ways of using, and allowing others, to use the data.

### Electronic supplementary material

Below is the link to the electronic supplementary material.

Supplementary Material 1

## Data Availability

Coronial data used in this study are not publicly available due to their sensitive nature. Therefore, the dataset supporting the findings in this study cannot be shared unless the person has obtained ethical approval due to the terms of the ethics agreement under which use of data was approved. For those interested in receiving access to data, please contact the National Coronial Information System (NCIS) via email: ncis@ncis.org.au. If you would like to discuss data access further, please contact the corresponding author via email (a.peden@unsw.edu.au).
