# Supplementary material for: Lessons learned through the 20-year development of a national fatal drowning database in Australia
Source: BMC Public Health. 2023 Aug 7;23:1499. doi: 10.1186/s12889-023-16392-2 (PMC10408144; doi:10.1186/s12889-023-16392-2)
Supplement: Supplementary file 1 — Supplementary Material 1 [file 12889_2023_16392_MOESM1_ESM.docx]

Supplementary Table 1: Complete list of variables housed within the National Fatal Drowning Database

| Number | Variable name |
| --- | --- |
| 1 | NCIS Number |
| 2 | State of Death |
| 3 | State or Territory of Coronial File |
| 4 | Fin Year Numeric |
| 5 | Status of Case |
| 6 | Checked Date |
| 7 | Sex |
| 8 | Age months and years |
| 9 | Age in Years |
| 10 | Age in whole years |
| 11 | Age_Months |
| 12 | Age Groups in 11 levels |
| 13 | Age Groups by 5 year bands |
| 14 | Life stage |
| 15 | Children 0 - 14 years |
| 16 | Born overseas YN |
| 17 | Country of Birth |
| 18 | Australian resident or overseas visitor |
| 19 | Time_in_country |
| 20 | Visa |
| 21 | Aboriginal and Torres Strait Islander (ATSI) Status |
| 22 | ATSI yes no |
| 23 | Medical Cause of Death |
| 24 | Other Medical Causes Identified |
| 25 | Underlying Medical Condition (Yes/No) |
| 26 | Medical_conidtion_known |
| 27 | Type of Underlying Medical Condition |
| 28 | Medical condition contributed |
| 29 | Name of major medical condition that contributed |
| 30 | Additional medical condiitons |
| 31 | Date of Death |
| 32 | Date of Incident |
| 33 | Date of Incident - Year |
| 34 | Date of Incident - Month |
| 35 | Date of Incident- Day |
| 36 | Day of week |
| 37 | Season of Incident |
| 38 | Financial Year |
| 39 | Time of Incident |
| 40 | Time of Incident Coded |
| 41 | Time Coded Bands |
| 42 | Resident Latitude |
| 43 | Resident Longitude |
| 44 | Residential Post Code |
| 45 | Residential LGA |
| 46 | Remoteness Classification of Residential Post Code |
| 47 | Recoded residential postcode remoteness |
| 48 | Country of Residential Postcode |
| 49 | Region of country of residence |
| 50 | Location Detailed |
| 51 | Location Name |
| 52 | Location Specific |
| 53 | RLSSA Location Coded |
| 54 | RLS Location Coded Numeric |
| 55 | RLS Inland waterway coding |
| 56 | Name of Lake |
| 57 | Name Of River |
| 58 | Swimming Pool Yes No |
| 59 | Private swimming pool (including outdoor spas and portables) |
| 60 | Public swimming pool |
| 61 | Communal swimming pool |
| 62 | Outdoor spa yes no |
| 63 | Bath Yes No |
| 64 | Time Location (Incident) Post Code |
| 65 | Incident Road Address |
| 66 | Incident Latitude |
| 67 | Incident Longitude |
| 68 | Driving distance between resident and incident road location (in km) |
| 69 | Driving distance grouped |
| 70 | Driving time between resident and incident road location (in minutes) |
| 71 | Driving time grouped (hours) |
| 72 | Visitor Status |
| 73 | Resident Address |
| 74 | Incident NCIS Address |
| 75 | Remoteness Classification of Time Location (Incident) Post Code |
| 76 | Incident region |
| 77 | Incident LGA 2011 Codes |
| 78 | Local Government Area for incident via NCIS |
| 79 | Activity Detailed |
| 80 | Activity Specific |
| 81 | RLSSA Activity Coded |
| 82 | Activity numeric |
| 83 | Unintentional or water transport |
| 84 | Boating or watercraft |
| 85 | Type of boat or watercraft |
| 86 | Lifejacket |
| 87 | Skipper or Passenger |
| 88 | Rescue Rock Fishing |
| 89 | Multiple Fatality Event |
| 90 | Multiple Fatality Incident Number |
| 91 | Number of victims per multiple fatality |
| 92 | Flood Related |
| 93 | Swimming Ability of Deceased |
| 94 | Swimming ability (coded) |
| 95 | Alcohol (Yes/No) |
| 96 | Blood Alcohol Content (BAC) |
| 97 | Alcohol relevance? |
| 98 | Drugs (Yes/No) |
| 99 | Type of Drug Known |
| 100 | Drug Legal (Yes/No) |
| 101 | Drug Type |
| 102 | Drug Type and Reading |
| 103 | Drugs relevant? |
| 104 | Notes (General) |
| 105 | Other Information - to be deleted |
| 106 | Status of Autopsy/ Finding Document/ Police Report/ Toxicology Report |
| 107 | Finding available |
| 108 | Autopsy available |
| 109 | Toxicology available |
| 110 | Police report available |
| 111 | Coronial Recommendation (Yes/No) |
| 112 | Coronial findings or recommendations |
| 113 | Coronial findings or recommendations (coded) |
| 114 | Employment Status |
| 115 | Usual Occupation |
| 116 | Marital Status |
| 117 | Date of Birth |
| 118 | Nationality |
| 119 | ICD-10 Level 1 |
| 120 | ICD-10 Level 2 |
| 121 | ICD-10 Level 3 |
| 122 | ICD-10 Level 4 |
| 123 | ICD-10 Level 5 |
| 124 | ICD-10 Level 6 |
| 125 | ICD-10 Level 7 |
| 126 | ICD-10 Level 8 |
| 127 | ICD10-Level 9 |
| 128 | ICD10-Level10 |
| 129 | ICD-10 Underlying Cause of Death |
| 130 | Contributing factors |
| 131 | ASGC Code for Incident Location |
| 132 | Incident RA |
| 133 | Residential RA |
| 134 | Local Council Recode |
| 135 | Incident LGA |
| 136 | AWSS people category |
| 137 | AWSS population category ATSI |
| 138 | AWSS population category multicultural |
| 139 | AWSS population category regional and remote |
| 140 | AWSS places category |
| 141 | AWSS activities category |
| 142 | AWSS risk factor category |
| 143 | Shallow Water Blackout |
| 144 | Heart medications and blood pressure |
| 145 | Narcotics |
| 146 | Pain |
| 147 | Tranq |
| 148 | Antidepressants |
| 149 | Other |
| 150 | Epilepsy yes or no |
| 151 | Epilepsy |
| 152 | Therapeutic_level |
| 153 | Toxic_level |
| 154 | Alcohol and drugs combined relevant |
| 155 | Alcohol/Drugs Contributing |
| 156 | Austim_Spectrum |
| 157 | infectious and parasitic diseases |
| 158 | Neoplasms |
| 159 | Diseases of the blood and blood forming organs |
| 160 | Endocrine, nutritional and metabolic diseases |
| 161 | Mental and behavioural problems |
| 162 | Disease of the Nervous System |
| 163 | Disease of the eye and adnexa |
| 164 | Disease of the circulatory system |
| 165 | Disease of the respiratory system |
| 166 | Disease of the digestive system |
| 167 | Disease of the skin and subcutaneous tissue |
| 168 | Disease of the musculoskeletal system and connective tissue |
| 169 | Disease of the genito_urinary system |
| 170 | Congenital malformations, deformations and chromosomal abnormalities |
| 171 | Disease of the ear and mastoid |
| 172 | Other signs, symptoms and conditions |
| 173 | Body Mass Index |
| 174 | Body Mass Index by Category |
| 175 | IRSAD for residential postcode |
| 176 | School holiday |
| 177 | Public Holiday by residence |
| 178 | Public Holiday by location |
| 179 | Public holiday long weekend |
| 180 | Holiday |
| 181 | statistical area 2 for incident location |
| 182 | statistical area 3 for incident location |
| 183 | statistical area 4 for incident location |
| 184 | Remoteness Area for incident location |
| 185 | statistical area 2 for victim residence |
| 186 | statistical area 3 for victim residence |
| 187 | statistical area 4 for victim residence |
| 188 | Local Government Area for victim residence via NCIS |
| 189 | Remoteness Area for victim residence |
| 190 | Older people falls |
| 191 | Mobility notes |
| 192 | History of falls |
| 193 | Visual impairment |
| 194 | Mobility coded |
| 195 | Environmental hazards notes |
| 196 | Environmental hazard coded |
| 197 | Other notes |
| 198 | Mobility aid coded |
| 199 | Clothing coded |
| 200 | Footwear condition coded |
| 201 | Was CPR Enacted |
| 202 | If CPR performed by who |
| 203 | CPR administered (by who, what type, equipment used, when started, how long) |
| 204 | Treatment administered |
| 205 | Treatment administered (yes or no) |
| 206 | Private_public_dam |
| 207 | dam_drown_accident |
| 208 | Dam_working |
| 209 | Search_body |
| 210 | search_for_body |
| 211 | alone_during_incident |
| 212 | if_no_lost_visability |
| 213 | safe_play_area |
| 214 | agriculture_industry |
| 215 | Type of swimming pool (coded) |
| 216 | Home pool yes or no (excluding portables) |
| 217 | Portable Pool - yes no |
| 218 | Pool Ownership |
| 219 | Swimming Pool Fenced |
| 220 | Supervision |
| 221 | Supervision coded |
| 222 | Description_of_fence |
| 223 | Pool Means of Access |
| 224 | Were Emergency Services Called |
| 225 | Type of swimming pool (size, shape) |
| 226 | Size of swimming pool (coded) |
| 227 | Method of access |
| 228 | Method of access (coded) |
| 229 | Location of incident within pool (depth, lane) |
| 230 | Location of incident coded |
| 231 | Ownership and management of pool |
| 232 | Number of patrons (including low patronage) |
| 233 | Number of patrons (coded) |
| 234 | Signage (content, visibility) |
| 235 | RLSSA references (Keep Watch, Watch Around Water, GSPO) |
| 236 | Responsibility for supervision |
| 237 | Supervision description |
| 238 | Supervision Absent Time |
| 239 | Supervision Absent Estimate |
| 240 | Supervision by parent or carer (who, absent for how long) |
| 241 | Supervisor (relation to deceased) |
| 242 | Activity in pool |
| 243 | Observed or found by |
| 244 | Observed or found by (coded) |
| 245 | Retrieved by (coded) |
| 246 | Lifeguards present (yes or no) |
| 247 | Supervision by lifeguard (number, location, activity, demographics, training) |
| 248 | If yes, number of lifeguards present |
| 249 | Emergency services contact (who called) |
| 250 | Emergency services contacted (yes or no) |
| 251 | If emergency services contacted, by who |
| 252 | Did police attend the scene or hospital? |
| 253 | Was WHS-related organisation involved? |
| 254 | How were WHS involved? |
| 255 | State of patient (airway, skin colour, state of consciousness) |
| 256 | Regional_Only |
| 257 | Inland_waterway_Recoded |
| 258 | Checked_LSV |
| 259 | Checked_WA |
